# Obstetrical Society

**Published:** 1898-12-24

**Authors:** 


### THE CLINICAL SOCIETY.

### OBSTETRICAL SOCIETY.

Dr. Herbert Spencer showed one case and mentioned another, which were usefully contrasted with one another. The first was a small multilocular dermoid tumour of the right ovary, which, being incarcerated in the pelvis, he had removed by laparotomy at the fourth month of pregnancy. Pain which had been present at once ceased on the removal of the tumour, and the patient had five months later been successfully delivered of a living child at full term. The second was a small ovarian dermoid tumour, which Dr. Spencer showed for another practitioner. It had been incarcerated in the pelvis at the time of labour. Forceps and afterwards version had been employed, with the result that the tumour was ruptured, and the patient died of septic peritonitis three days after delivery. Commenting upon this case, Dr. John Phillips mentioned a case in which the cyst had obstructed labour, and had necessitated its incision and suturing to the vaginal walls before labour could be completed. A year later urgent symptoms led to operation, when a suppurating dermoid was found adherent to the scar in the vagina. Dr. Phillips had recently successfully removed an impacted dermoid complicating early pregnancy. Dr. Giles also mentioned a case of an ovarian dermoid which he had removed during pregnancy without precipitating labour.

